# Non genomic loss of function of tumor suppressors in CML: BCR-ABL promotes IκBα mediated p53 nuclear exclusion

**DOI:** 10.18632/oncotarget.4611

**Published:** 2015-07-23

**Authors:** Sabrina Crivellaro, Cristina Panuzzo, Giovanna Carrà, Alessandro Volpengo, Francesca Crasto, Enrico Gottardi, Ubaldo Familiari, Mauro Papotti, Davide Torti, Rocco Piazza, Sara Redaelli, Riccardo Taulli, Angelo Guerrasio, Giuseppe Saglio, Alessandro Morotti

**Affiliations:** ^1^ Department of Clinical and Biological Sciences, University of Turin, Orbassano, Italy; ^2^ Division of Pathology, Department of Oncology, University of Turin at St Luigi Hospital, Torino, Italy; ^3^ Department of Health Sciences, University of Milano-Bicocca, Monza, Italy; ^4^ Department of Oncology, University of Turin, Turin, Italy

**Keywords:** NF-κB, IκBα, chronic myeloid leukemia, p53, tumor suppressor

## Abstract

Tumor suppressor function can be modulated by subtle variation of expression levels, proper cellular compartmentalization and post-translational modifications, such as phosphorylation, acetylation and sumoylation. The non-genomic loss of function of tumor suppressors offers a challenging therapeutic opportunity. The reactivation of a tumor suppressor could indeed promote selective apoptosis of cancer cells without affecting normal cells. The identification of mechanisms that affect tumor suppressor functions is therefore essential. In this work, we show that BCR-ABL promotes the accumulation of the NFKBIA gene product, IκBα, in the cytosol through physical interaction and stabilization of the protein. Furthermore, BCR-ABL/IκBα complex acts as a scaffold protein favoring p53 nuclear exclusion. We therefore identify a novel BCR-ABL/IκBα/p53 network, whereby BCR-ABL functionally inactivates a key tumor suppressor.

## INTRODUCTION

The involvement of tumor suppressors in cancer pathogenesis has been extensively revised over the last years. Originally, Knudson proposed that tumor suppressors play a role in tumorigenesis when both alleles are genetically impaired, one through point mutation and one through deletion [1]. Several data collected in non hereditary tumors and in murine models have clearly demonstrated that tumor suppressors' involvement in cancer is a much more complex process [2]. In particular, tumor suppressor function can be modulated by subtle variation in protein expression levels, proper cellular compartmentalization and post-translational modifications of the protein, such as phosphorylation, acetylation and sumoylation [3, 4]. Chronic Myeloid Leukemia is a myeloproliferative disorder driven by the translocation t(9;22) which codes for the chimeric protein BCR-ABL [5–10]. Chronic Myeloid Leukemia has always been considered as an unique disease because it was referred as a ‘single hit’ cancer [11]. This interpretation is supported by the observation that BCR-ABL alone is sufficient to induce the rapid onset of a leukemic phenotype in several murine models, without the need of additional genetic lesions [12]. Furthermore, no tumor suppressors have been reported mutated or deleted in the chronic phase of the disease, strongly supporting this unique biological feature of CML pathogenesis compared to other cancers. The revised model of tumor suppressor role in cancer suggests that BCR-ABL-mediated leukemogenesis could be associated with functional non genomic loss of tumor suppressors [2]. In this respect, PP2A and PTEN have already been described as BCR-ABL inhibited tumor suppressors [13–18]. Here, we demonstrate that BCR-ABL promotes the formation of a ternary complex with IκBα and p53 in the cytoplasm causing loss of p53 tumor suppressive nuclear pool.

## RESULTS AND DISCUSSION

**Figure 1:**
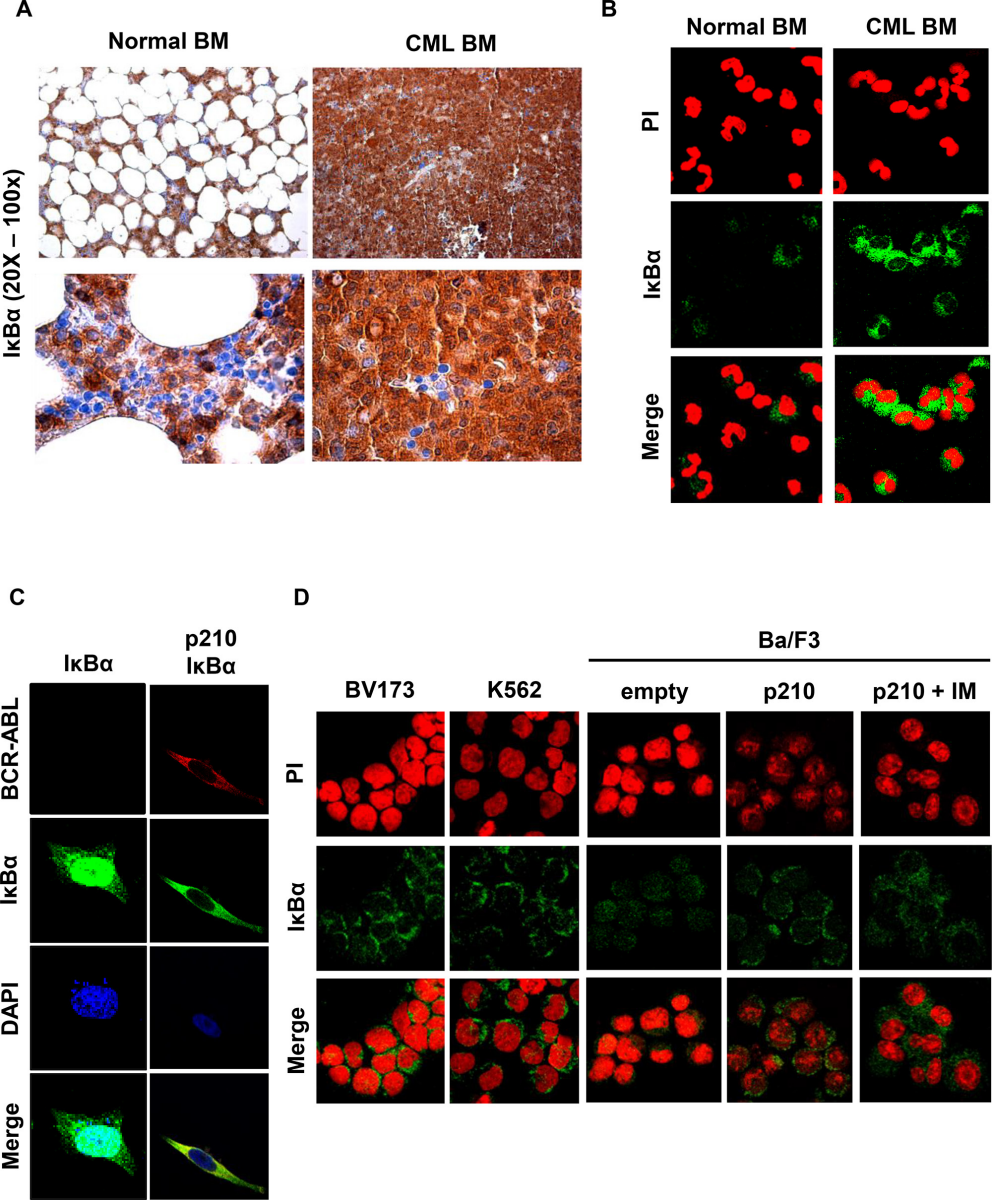
IκBα in BCR-ABL positive cells **A.** Representative immunohistochemistry using an anti-IκBα antibody performed on paraffin-embedded bone marrow from a representative CML or normal bone marrow. IHC was performed on 4 CML chronic phase samples with comparable results. **B.** Representative immunofluorescence on normal and CML primary bone marrow cells were performed to detect endogenous IκBα (green signal). Nuclei were stained with propidium iodide (red). Immunofluorescence was performed on 20 chronic phase CML samples and 3 normal bone marrow samples with comparable results. **C.** HeLa cells were transfected with BCR-ABL and IκBα plasmids. Immunofluorescence staining of IκBα (green) and BCR-ABL (red) was performed to detect IκBα localization. Nuclei were stained with DAPI. **D.** Immunofluorescence on the BV173 and K562 CML cell lines and on parental Ba/F3 and Ba/F3 p210 BCR-ABL cells was performed to detect IκBα (green signal). Nuclei were stained with propidium iodide (red). When indicated, cells were treated for 6 hours with 10 μM of imatinib.

While investigating the cellular compartmentalization of IκBα in CML formalin-fixed paraffin-embedded specimens, we observed that IκBα was highly expressed and predominantly retained into the cytosol of myeloid progenitor cells when compared to normal bone marrow cells (Figure 1A). Interestingly, erythroid precursors do not express IκBα protein (Figure 1A). Similarly, IκBα immunofluorescence on CML primary cells showed that IκBα is expressed predominantly in the cytoplasm (Figure 1B). To evaluate whether IκBα cellular compartmentalization is regulated by BCR-ABL, we expressed BCR-ABL and myc-tag-IκBα in HeLa cells. These cells were chosen because the large amount of cytoplasm allows to easily study cellular compartmentalization. As shown in Figure 1C, exogenous IκBα maintains a diffuse cellular compartmentalization in parental cell line, while expression of BCR-ABL promotes its nuclear exclusion. Notably, BCR-ABL and IκBα appeared also to co-localize (Figure 1C).

To better evaluate IκBα localization in CML cell lines, IκBα immunofluorescence was performed in the CML cell lines BV173 and K562 and in the Ba/F3 p210 BCR-ABL cells [19]. As shown in Figure 1D, IκBα is mostly expressed in the cytosol of the BV173 and K562 cell lines. Parental Ba/F3cells expressed IκBα both in the nucleus and in the cytosol, while Ba/F3 p210 BCR-ABL are characterized by IκBα nuclear exclusion (Figure 1D). Similar results were observed in the BCR-ABL-32D cell line (data not shown). Notably, imatinib treatment does not significantly revert IκBα cellular localization (Figure 1D).

**Figure 2:**
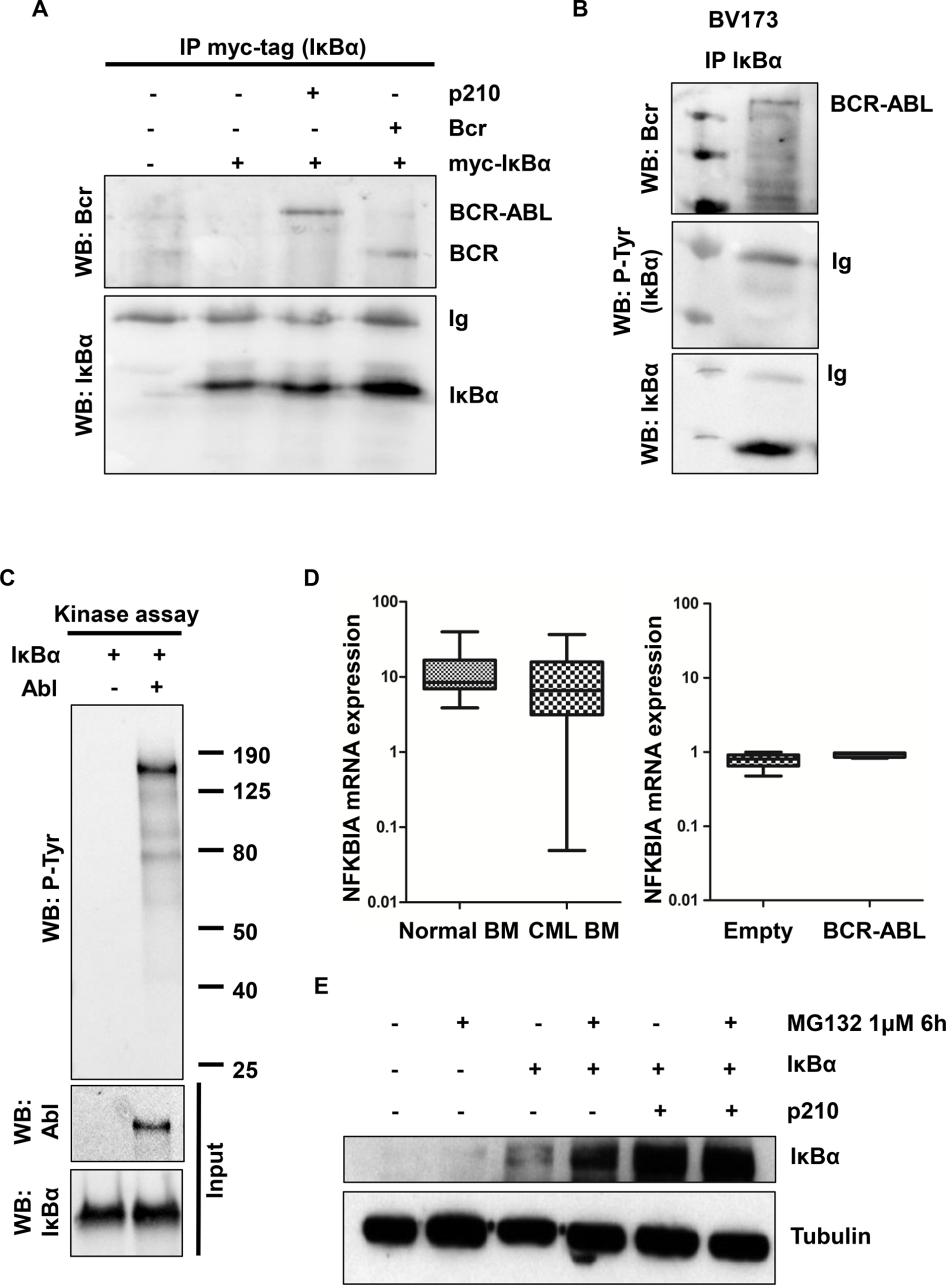
IκBα interactions and phosphorylation in BCR-ABL transfected cells **A.** HEK293T cells were transfected with the indicated plasmids and assessed by immunoprecipitation with myc-tag antibody (IκBα) and western blot. **B.** BV173 lysates were immunoprecipitated with IκBα antibody and western blot **C.** *In vitro* kinase assay with purified ABL and IκBα proteins was performed. P-Tyr: phospho-tyrosine. **D.** qRT-PCR analysis of NFKBIA (IκBα) performed on normal and CML bone marrow samples and on HEK293T BCR-ABL and empty vector transfected cells. **E.** BCR-ABL and IκBα-transfected HEK293T cells were incubated for 6 hours with 1 μM of MG132. Western blot analysis was performed to evaluate IκBα protein level. Tubulin was used as loading control.

The observation that BCR-ABL and IκBα co-localized in transfected HeLa cells (Figure 1C) suggests that BCR-ABL could interact with IκBα. To test this hypothesis, HEK293T cells, upon transfection with BCR-ABL and IκBα vectors, were lysed and immunoprecipitation with myc-tag antibody was performed. As shown in Figure 2A, myc-tag-IκBα interacts with BCR-ABL. To map the BCR-ABL region that interacts with IκBα, we transiently transfected a BCR expressing vector together with myc-tag-IκBα in the HEK293T cell line. As shown in Figure 2A, BCR-ABL physically interacts with IκBα through the BCR portion of the chimeric protein. Notably, in the CML BV173 cell line, endogenous BCR-ABL interacts with endogenous IκBα (Figure 2B). Due to the intrinsic nature of BCR-ABL as a tyrosine kinase, we next sought to investigate whether BCR-ABL promotes IκBα tyrosine phosphorylation. To this aim, we performed a kinase assay with purified IκBα protein. As shown in figure 2C, IκBα is not phosphorylated by purified ABL *in vitro*. Although BCR-ABL does not directly phosphorylate IκBα, we next explored whether BCR-ABL could indirectly tyrosine phosphorylate IκBα *in vivo*. We performed immunoprecipitation of myc-tag-IκBα in BCR-ABL positive cells, and we assessed the phospho-tyrosine status of IκBα by western immunoblot. IκBα does not appear to be phosphorylated *in vivo* (data not shown). This observation was also observed in the BV173 cell line, where immunoprecipitated IκBα does not hybridize with anti-phosphotyrosine antibody (Figure 2B). All together these data suggest that IκBα behaves as a scaffold protein in BCR-ABL signaling. Next, we investigated if BCR-ABL regulates IκBα expression and stability. To evaluate IκBα mRNA expression levels, we performed qRT-PCR in BCR-ABL cell lines and primary CML cells. As shown in Figure 2D, there were no statistically significant differences between normal bone marrow and CML chronic phase, and between empty vector transfected cells and BCR-ABL ones, suggesting that IκBα mRNA levels are not regulated by BCR-ABL at least at the steady state. We then performed a stability assay of IκBα protein. HEK293T cells transfected with IκBα and with Bcr-Abl were incubated for 6 hours with the proteasome inhibitor MG-132. BCR-ABL appeared to regulate IκBα protein stability at a post-translational level (Figure 2E).

**Figure 3:**
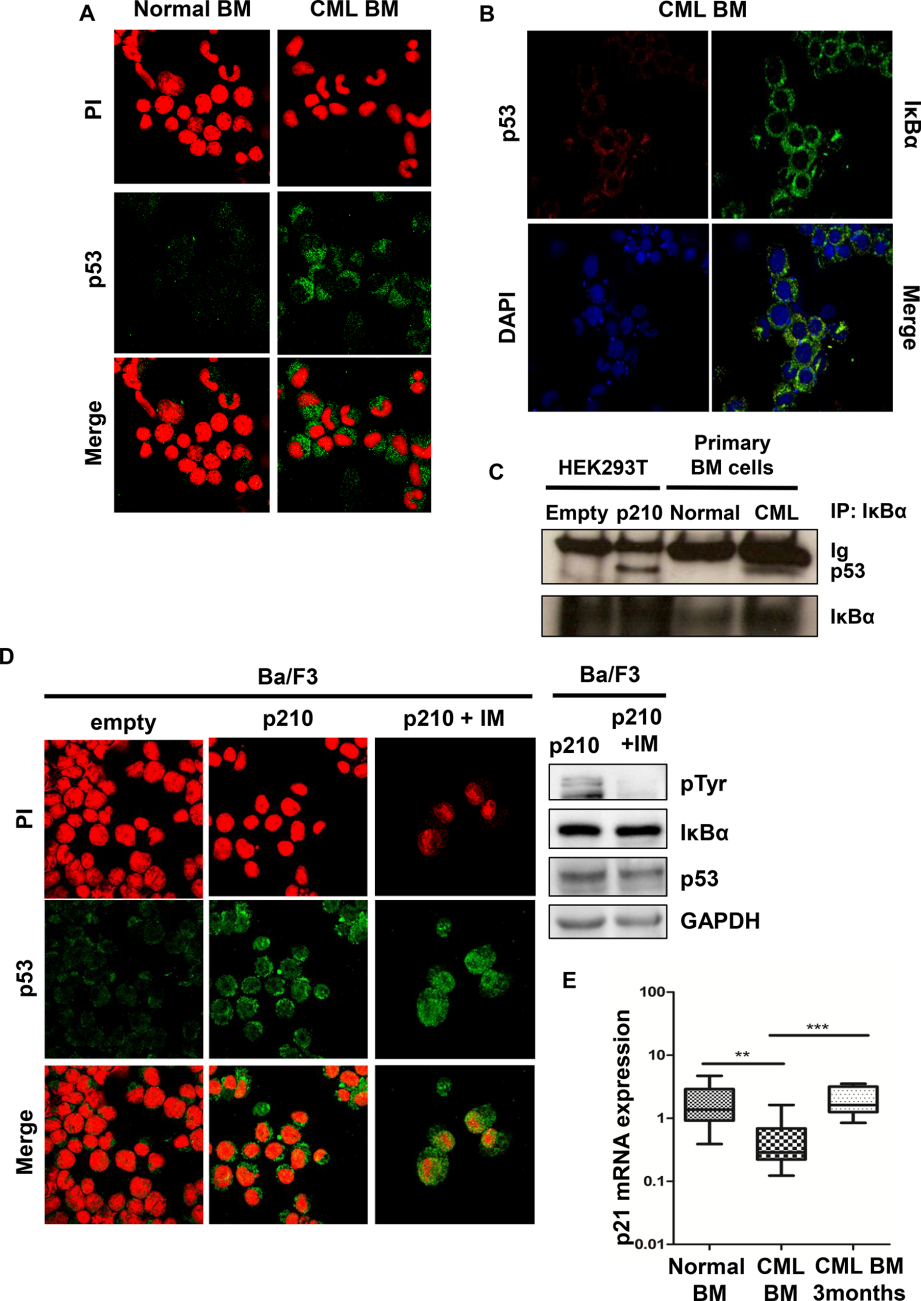
p53 in BCR-ABL positive cells **A.** Representative immunofluorescence on normal and CML primary bone marrow cells were performed to detect endogenous p53 (green signal). Nuclei were stained with propidium iodide (red). Immunofluorescence was performed on 20 chronic phase CML samples and 3 normal bone marrow samples with comparable results. **B.** Primary CML samples were stained with anti-IκBα (green) and anti-p53 (red) antibodies to assess co-localization of p53 with IkBα. **C.** Immunoprecipitations with IκBα antibody were performed on HEK293T transfected cells and on primary BM cells. **D.** Immunofluorescence on Ba/F3 parental and Ba/F3 p210 BCR-ABL cell line was performed to detect p53 (green signal). Nuclei were stained with propidium iodide (red). Right panel: Western blot on Ba/F3 p210 BCR-ABL imatinib-treated cells. When indicated, cells were treated with imatinib for 6 hours at a concentration of 10 μM. **E.** qRT-PCR analysis of p21 performed on normal and CML bone marrow at the diagnosis (**p < 0.05) and after 3-months of imatinib treatment (***p < 0.005).

IκBα is mostly studied for its inhibitory activity toward NF-κB, through the interaction with the NF-κB subunit p65 [21, 22]. IκBα is a shuttling protein that moves into the nucleus, where it binds to active NF-κB and inhibits its transcriptional activity by removing it from the nucleus. NF-κB was extensively studied in the pathogenesis of CML [23–29]. IκBα was also shown to bind to p53, which shares with p65 NF-κB a similar tridimensional structure [30–32]. TP53 is a tumor suppressor that is involved in several cellular processes, like senescence, cell cycle inhibition, apoptosis and DNA damage repair [33, 34]. In CML, p53 was shown to be involved in the progression into the blast crisis, where almost 20% of the patients display mutated TP53, while TP53 was never found mutated/deleted in the chronic phase of the disease [35]. Therefore, we hypothesized that IκBα could also promote p53 sequestration from the nucleus in CML. To this aim, we performed p53 immunofluorescence in primary CML cells. As shown in Figure 3A, p53 is retained into the cytosol of CML cells. Notably, nuclear excluded p53 was found to co-localize with IκBα in the cytoplasm (Figure 3B). To better validate these observations, we confirmed that IκBα co-immunoprecipitates with p53 in the presence of BCR-ABL both in transfected cells and in primary CML bone marrow cells (Figure 3C).

All together these data indicate that BCR-ABL promotes cytoplasmic sequestration of the IκBα-p53 complex. Previous works have clearly demonstrated that the IκBα-p53 complex is associated with the inhibition of p53 [30–32]. Notably, TNFα and the tyrosine kinase inhibitor staurosporine were shown to disrupt this complex, although the mechanism was unknown [30]. Our observations in primary CML samples and cell lines suggest that BCR-ABL promotes the sequestration and inactivation of p53 in the cytoplasm through the interaction with IκBα. The inactivation of p53 through sequestration and/or delocalization in the cytoplasm could harbor important therapeutic options. To test this hypothesis, we treated Ba/F3 p210 BCR-ABL cells with imatinib and we assessed the cellular compartmentalization of p53. Importantly, imatinib promotes p53 relocalization into the nucleus (Figure 3D) and apoptosis/growth arrest induction (data not shown).

To better evaluate whether the delocalization of p53 is associated with impairment of its function, we measured p21 mRNA expression in CML samples. TP53 is indeed known to regulate p21 expression [36]. As shown in Figure 3E, p21 mRNA levels are markedly reduced in CML primary cells, compared to normal bone marrow while imatinib treatment promotes p21 levels restoration.

**Figure 4:**
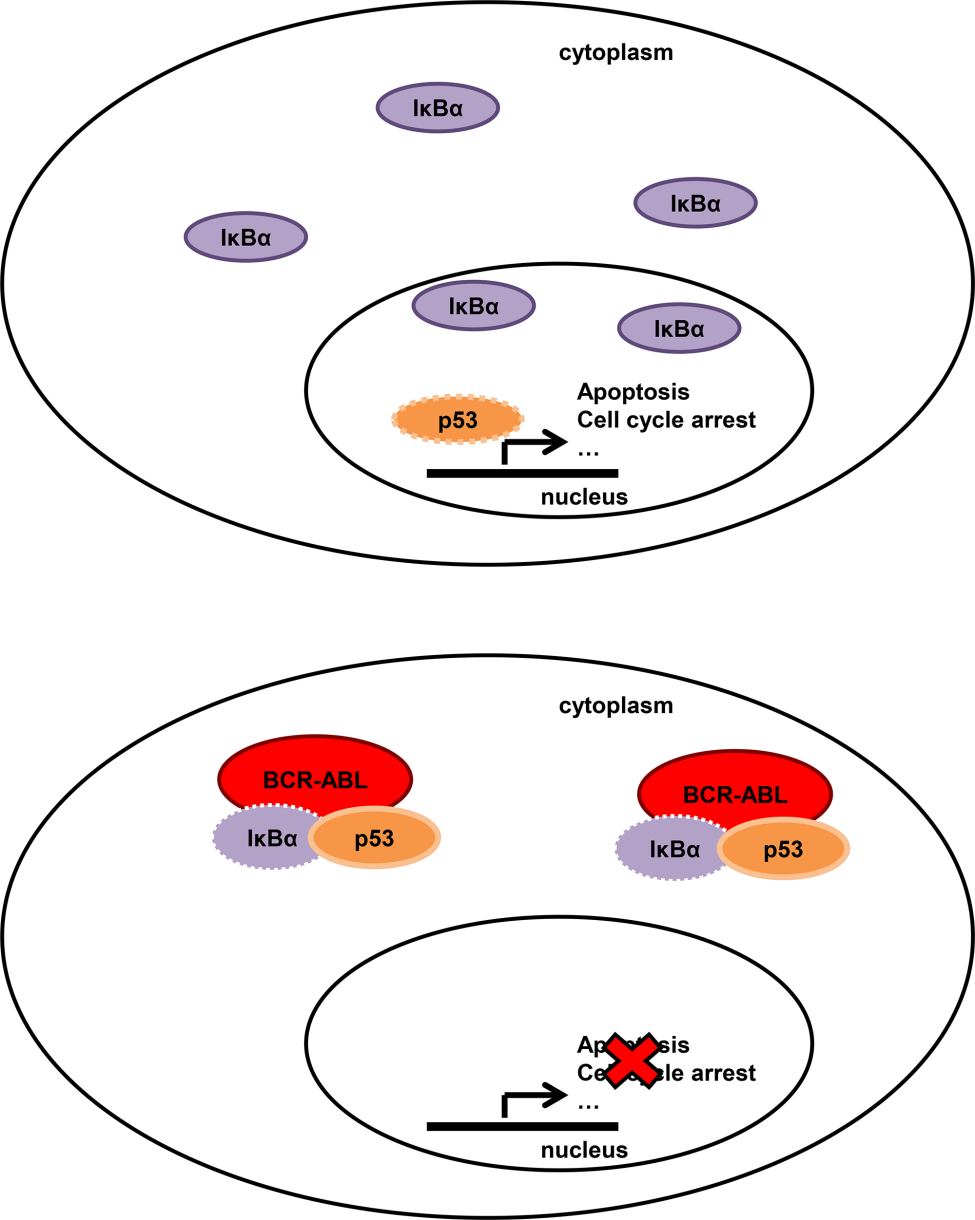
Model of BCR-ABL-IκBα-p53 network in normal (upper panel) and BCR-ABL positive cells (bottom panel) In normal cells IκBα can shuttle from the cytoplasm to the nucleus to remove NF-κB and p53 from the DNA. In BCR-ABL positive cells, IκBα is mostly cytoplasmic, interacts with BCR-ABL and with p53. The BCR-ABL/IkBα network enables p53 to translocate into the nucleus and therefore functionally inactivate its tumor suppressive functions.

In summary, we have demonstrated that in Chronic Myeloid Leukemia BCR-ABL physically interacts with IκBα, promotes its cytoplasmic localization, and favors the maintenance of the IκBα/p53 complex into the cytoplasm. As a consequence, BCR-ABL negatively regulates the pro-apoptotic and growth arrest potentials of p53 (Figure 4).

Our work leads to the following conclusions. Firstly, BCR-ABL-mediated tumorigenesis does not only depend on the direct activation of pathways involved in the regulation of cell growth and survival, but also on the ability of BCR-ABL to counteract pathways that suppress proliferation and survival. In line with these considerations, BCR-ABL was already shown to inactivate the tumor suppressors PP2A, PTEN and others [13, 15–17]. Here, we provide evidences that BCR-ABL/IκBα complex affects the tumor suppressive functions of p53.

Secondary, our work further highlights the relevance of the IκBα-p53 complex in cancer biology. This interaction has been already described although the mechanisms of regulation and the biological relevance were almost unknown [30–32]. Our data suggest that IκBα is able to orchestrate two essential factors in tumorigenesis. From one side IκBα negatively regulates the NF-κB subunit p65 and from the other p53. Therefore, we suggest that oncogenes could switch the balance of this interaction, favoring inactivation of p53. However, our data could not completely explain the mechanisms of IκBα-p53 formation and regulation. In a previous report, the IκBα-p53 complex is disrupted by treatment with the tyrosine kinase inhibitor straurosporine, suggesting that IκBα-p53 complex is regulated by tyrosine kinases. Imatinib is also able to promote IκBα-p53 disruption downstream of BCR-ABL, although we did not observe a direct IκBα tyrosine phosphorylation by BCR-ABL. We therefore speculate that other kinases, downstream of BCR-ABL, or BCR-ABL kinase independent pathways, could be involved in the formation and regulation of the IκBα-p53 complex.

Finally, our data imply that the BCR-ABL/IκBα/p53 network harbors therapeutic implications. Storing an active and genetically wild-type tumor suppressor could indeed offer the chance to design therapies to reactivate p53 with dramatic cancer selective apoptosis induction.

## MATERIALS AND METHODS

### Cells, primary human samples and pharmacological treatments

HEK293T, HeLa, K562, BV173 cells were obtained from ATCC and cultured according to vendor. Parental Ba/F3 and Ba/F3 p210 BCR-ABL were previously reported [19]. Twenty primary chronic phase CML and three normal bone marrow samples were collected at the San Luigi Hospital (Orbassano, Italy) with informed consent of patients at the time of diagnosis. Project was reviewed by the institutional Ethical Committee (code #10/2013). Cell lines were incubated with 10 μM Imatinib mesylate (Novartis) for 6 hours and with 1 μM MG132 (Selleckchem) for 6 hours.

### Plasmids and transfection

NFκBIA plasmid [20] was cloned into myc-tagged pRK5 plasmid [15]. BCR-ABL vector was described elsewhere [15]. BCR vector was cloned in pcDNA3.1 plasmid. Transient transfection was performed with X-treme GeneHP (Roche) reagent according to manufacturer's protocol.

### Western immunoblot and immunoprecipitation

Proteins were extracted with a buffer containing 150 mM NaCl, 1 mM EDTA, 50 mM HEPES (pH 7.5), 1% Triton X-100 and 10% glycerol. For immunoprecipitations, lysates were precleared with normal mouse IgG antibody (Santa Cruz, #2025) or normal rabbit IgG (Santa Cruz, #2027)and Protein A/G-PLUS-Agarose (Santa Cruz, # sc-2002) for 1 hour. Then lysates were incubated with antibody overnight at 4°C on a rotator. Protein A/G-PLUS-Agarose beads were added and incubated for 2 hours at 4°C with rotation. Agarose beads bound with immunoreactive complexes were washed four times with cold co-IP buffer. Following the final wash, immunoprecipitated complexes were eluted with 30 μl of 2x sample buffer, boiled for 5 min and analyzed using SDS-PAGE. Equal amounts of proteins were denatured in Laemmli sample buffer at 95°C for 5 min, resolved by SDS–PAGE on 8% gels and subsequently transferred to a nitrocellulose membrane. Immunoblotting was carried out using antibodies against IκBα (Cell signaling #4814), myc-tag (Cell signaling #2276), p53 (Santa Cruz sc-98), Bcr (Cell signaling #3902), P-Tyr (Santa Cruz sc-7020), c-Abl (Cell signaling #2862). Blots were scanned into the ChemiDoc XRS+ system (Biorad) and image acquisition and analysis was performed using ImageLab software (Biorad).

### Kinase assay

An *in vitro* kinase assay with purified full length ABL (Invitrogen, #P3049) and IκBα (BIOMOL, UW9975) proteins was performed as previously described [15].

### Immunofluorescence and immunohistochemistry

Immunofluorescence was performed by fixing cells with 4% PFA, permeabilizing them with 0.5% Triton X-100 and blocking with bovine serum albumin for 30 minutes. After blocking, cells were incubated with antibody at 1:100 at room temperature for 2 hours, followed by incubation with 1:500 secondary antibodies Alexa fluor-488 (Invitrogen # A-11078) and Alexa fluor-543 (Invitrogen # A-11030) at room temperature for 1 hour. Nuclei were stained with propidium iodide or DAPI for 5 minutes. Immunohistochemistry experiments were performed on formalin-fixed, paraffin-embedded tissues using anti-IκBα antibody (Cell Signaling, #4814) according to manufacturer's protocols.

### Real-time quantitative polymerase chain reaction

Total RNA was extracted using standard procedures. Quantitative Real-Time PCR (qRT-PCR) reactions and fluorescence measurements were performed using the 7300 RealTime PCR System (Applied Biosystem). For NFKBIA and p21 quantification, specific assays with on-demand primer/probe kits (assay ID 00355671_g1 for NFKBIA, Hs00355782_m1 for p21 and Hs00245445_m1 for ABL1) (Applied Biosystems, Foster City, CA, USA) were conducted according to the manufacturer's instructions. The NFKBIA and p21 Ct obtained by qRT-PCR was normalized with respect to the Ct of ABL and expressed as 2−ΔΔCt. Universal Human Reference RNA (Stratagene, #740000) was used to calibrate the assay.

### Statistical analysis

Statistical analysis was performed with GraphPad Prism v5.0d (GraphPad Software), using the Student t test. Significance was set at *P* < 0.05.
